# Encapsulation and Delivery of Therapeutic Phages

**DOI:** 10.1128/AEM.01979-20

**Published:** 2021-02-12

**Authors:** Belinda Loh, Vijay Singh Gondil, Prasanth Manohar, Fazal Mehmood Khan, Hang Yang, Sebastian Leptihn

**Affiliations:** a Zhejiang University-University of Edinburgh (ZJU-UoE) Institute, Zhejiang University, International Campus, Haining, Zhejiang, People’s Republic of China; b CAS Key Laboratory of Special Pathogens and Biosafety, Center for Biosafety Mega-Science, Wuhan Institute of Virology, Chinese Academy of Sciences, Wuhan, Hubei, People's Republic of China; c Department of Infectious Diseases, Sir Run Run Shaw Hospital, Zhejiang University School of Medicine, Hangzhou, People’s Republic of China; d University of Edinburgh Medical School, Biomedical Sciences, College of Medicine & Veterinary Medicine, The University of Edinburgh, Edinburgh, United Kingdom; e The Second Affiliated Hospital Zhejiang University (SAHZU), School of Medicine, Hangzhou, Zhejiang, People’s Republic of China; f University of the Chinese Academy of Sciences, Beijing, People's Republic of China; Centers for Disease Control and Prevention

**Keywords:** bacteriophage therapy, bacteriophages, delivery, delivery vehicles, encapsulation, nanoparticles

## Abstract

Delivery of therapeutic compounds to the site of action is crucial. While many chemical substances such as beta-lactam antibiotics can reach therapeutic levels in most parts throughout the human body after administration, substances of higher molecular weight such as therapeutic proteins may not be able to reach the site of action (e.g., an infection) and are therefore ineffective. In the case of therapeutic phages, i.e., viruses that infect microbes, to treat bacterial infections, this problem is exacerbated; not only are phages unable to penetrate tissues, but phage particles can be cleared by the immune system, and phage proteins are rapidly degraded by enzymes or inactivated by the low pH in the stomach. Yet, the use of therapeutic phages is a highly promising strategy, in particular for infections caused by bacteria that exhibit multidrug resistance. Clinicians increasingly encounter situations where no treatment options remain available for such infections where antibiotic compounds are ineffective. While the number of drug-resistant pathogens continues to rise due to the overuse and misuse of antibiotics, no new compounds are becoming available, as many pharmaceutical companies discontinue their search for chemical antimicrobials. In recent years, phage therapy has undergone massive innovation for the treatment of infections caused by pathogens resistant to conventional antibiotics. While most therapeutic applications of phages are well described in the literature, other aspects of phage therapy are less well-documented. In this review, we focus on the issues that are critical for phage therapy to become a reliable standard therapy and describe methods for efficient and targeted delivery of phages, including their encapsulation.

## Abstract (precis)

Delivery of therapeutic compounds to the site of action is crucial. While many chemical substances such as beta-lactam antibiotics can reach therapeutic levels in most parts throughout the human body after administration, substances of higher molecular weight such as therapeutic proteins may not be able to reach the site of action (e.g., an infection) and are therefore ineffective.

## INTRODUCTION

In 2017, the World Health Organization issued a report, defining the most dangerous antibiotic-resistant bacteria, the so-called ESKAPE group (1). This acronym describes resistant strains of Enterococcus faecium, Staphylococcus aureus, Klebsiella pneumoniae, Acinetobacter baumannii, Pseudomonas aeruginosa, and *Enterobacter* species, bacteria that can cause life-threatening diseases in both community- and hospital-acquired infections (2). These strains are almost “invincible superbugs,” as limited or no options are available for treatment, thus causing serious health care problems. Due to the overuse and misuse of antibiotics, an increasing number of resistant bacteria are being isolated from health care settings and the environment, where the rapid exchange of genetic elements and resistance genes among bacterial classes foster the spread of antimicrobial resistance (AMR). With the strategic financial decisions made by many global players in the pharmaceutical industry to discontinue or outsource discovery programs for novel antibiotics, the rise of antibiotic-resistant bacteria requires alternative treatment options to be developed (3, 4). One of the most promising strategies is phage therapy, where bacteriophages (or phages) are employed against bacterial pathogens (5, 6). This antibacterial therapy is currently undergoing a renaissance after a brief success a century ago, which was quickly (almost) abandoned for the triumphant chemical antibiotic “warfare” that now seems to have reached an impasse (7). Bacteriophages have been gaining increasing attention in recent years, especially due to their tremendous therapeutic potential against multidrug-resistant bacteria (8). The general safety of therapeutic bacteriophages prepared under good laboratory practice (GLP)/good manufacturing practice (GMP) conditions is one of the most important arguments for their use as treatments for antibiotic-resistant bacterial infections (9).

## BACTERIOPHAGES AND PHAGE THERAPY

Bacteriophages (also known as phages) are viruses that specifically infect bacteria and are considered the most abundant biological entities on earth. Phages can be classified based on their life cycle, being either “lytic” or “lysogenic” (10, 11). Immediately after infection by a lytic phage, the phage genome is replicated and proteins synthesized. After viral assembly, the host is then killed by lysis, a process facilitated by several viral proteins that destabilize the bacterial envelope (i.e., holins and endolysins), causing its rupture and the release of phage progeny (12). However, in the case of lysogenic phages, viral DNA is integrated into the host bacterial genome, which is only transcribed and translated for the synthesis of phage proteins by the host’s machinery under certain conditions, usually initiated by a trigger, such as DNA damage. Identical to lytic phages, the phage progeny is then released by host lysis, which eventually leads to the killing of the host bacterium (13). Few exceptions exist, such as filamentous phages (*Inoviridae*) that are produced while the host continues to grow and divide (14, 15).

Bacteriophages are considered one of the most promising alternative therapeutic agents replacing or complementing antibiotics for the treatment of multidrug-resistant (MDR) bacteria (16–18). In comparison to conventional antibiotics, bacteriophages are the only therapeutic agents whose concentration increases at the site of bacterial infection due to their “self-replicative” nature, i.e., their replication in the bacterial host (19). Therefore, administration of repeated doses of phages may not be required even though it is the common practice. In addition, phages remain in the body for a longer duration depending on the presence of the host bacterium (17, 20). Hence, the persistence of phages could reduce complications caused by side effects from conventional antibiotics and ultimately enhance treatment efficacy. The inherent physicochemical properties of bacteriophages allow bacteriophages to access sites of infection that may not be accessible by chemical compounds. Other properties, such as strong bactericidal activity and low intrinsic toxicity of bacteriophages, make phage therapy the favorable choice over conventional antibiotics (21–24). Phages usually infect a limited range of bacteria due to their high specificity and selectivity. This targeted nature of bacteriophages leaves normal microbiota intact and is one of the main advantages as a therapeutic agent, particularly important for immunocompromised patients and those with underlying conditions or allergies against chemical therapeutics (18).

## PHAGE DELIVERY SYSTEMS

Despite the numerous advancements in the preparation of phages for clinical applications, each route of administration represents its individual challenges (25). These include the stability of phage preparations, target-site-specific delivery, as well as the antibody-mediated inactivation of phages and their clearance by the reticuloendothelial system of the recipient (26–28). To optimize the efficacy and delivery of phages, formulations for therapeutic phages are under constant development (29–31).

Conventional phage preparations are liquid, comprised of medium supernatant that has been simply cleared from cells by centrifugation or filtration. Such crude preparations contain bacterial products, potentially including exotoxins, but also endotoxins such as lipopolysaccharide (LPS) from the lysed cells. However, several processes have been developed to allow the LPS-free production of liquid phage preparations (32–36). Liquid formulations are technically easy to produce and can generally be stored refrigerated for several years without a dramatic reduction in titer depending on the individual stability of the phage.

### Stabilized dry phage preparations (powders).

Lyophilization of proteinaceous compounds has had a long-standing history as a preservation method. Hence, it is no surprise to find that lyophilized phages are extensively used. Lyophilization or freeze-drying involves the dehydration of a phage-containing liquid, which is often supplemented with additives that prevent the inactivation of the phage by osmotic damage or phage particle aggregation caused by the dehydration process but is also beneficial to prevent inactivation during rehydration. Protectants include sugars (glucose, lactose, sucrose, trehalose, gluconate), amino acids (e.g., glutamate), proteins (e.g., lactoferrin), or more complex materials such as peptone, casein, or skimmed milk (37). Lyophilization produces particle sizes varying from nanometers to micrometers and retains the activity of the biotherapeutic material while also allowing their long-term storage.

An alternative to lyophilization is spray drying, which should be kept below 40°C to avoid denaturation and inactivation of the phage (38). In addition to the elevated temperatures, phages are also exposed to shear forces, which—similar to the delivery of phages as a spray—can lead to loss in titer (39–41). While these physical problems have a negative impact on the phage preparation, spray drying usually produces particles of 1 to 5 µm. The generation of such nano- or microparticles allows the production of phage powders that are easy to administer for the treatment of respiratory infections, as delivery via inhalers allows efficient nebulization (38, 40, 42–45).

The first successful therapy of a patient with cystic fibrosis was treated with S. aureus and P. aeruginosa phages via nebulization in combination with antibiotics (46). Other aerosolized powder-based phage preparations have been investigated in *in vitro* models for lung delivery. Lyophilized lactoferrin-based phage powder preparations have been investigated for the treatment of Burkholderia cepacia and P. aeruginosa infections (47). Agarwal and colleagues also showed that phage-loaded poly-lactic-co-glycolic acid microparticles were efficiently distributed throughout the lungs of mice and were more efficient than free phages in controlling the P. aeruginosa infection induced in a murine lung pneumonia model (48).

### Encapsulation.

**FIG 1:**
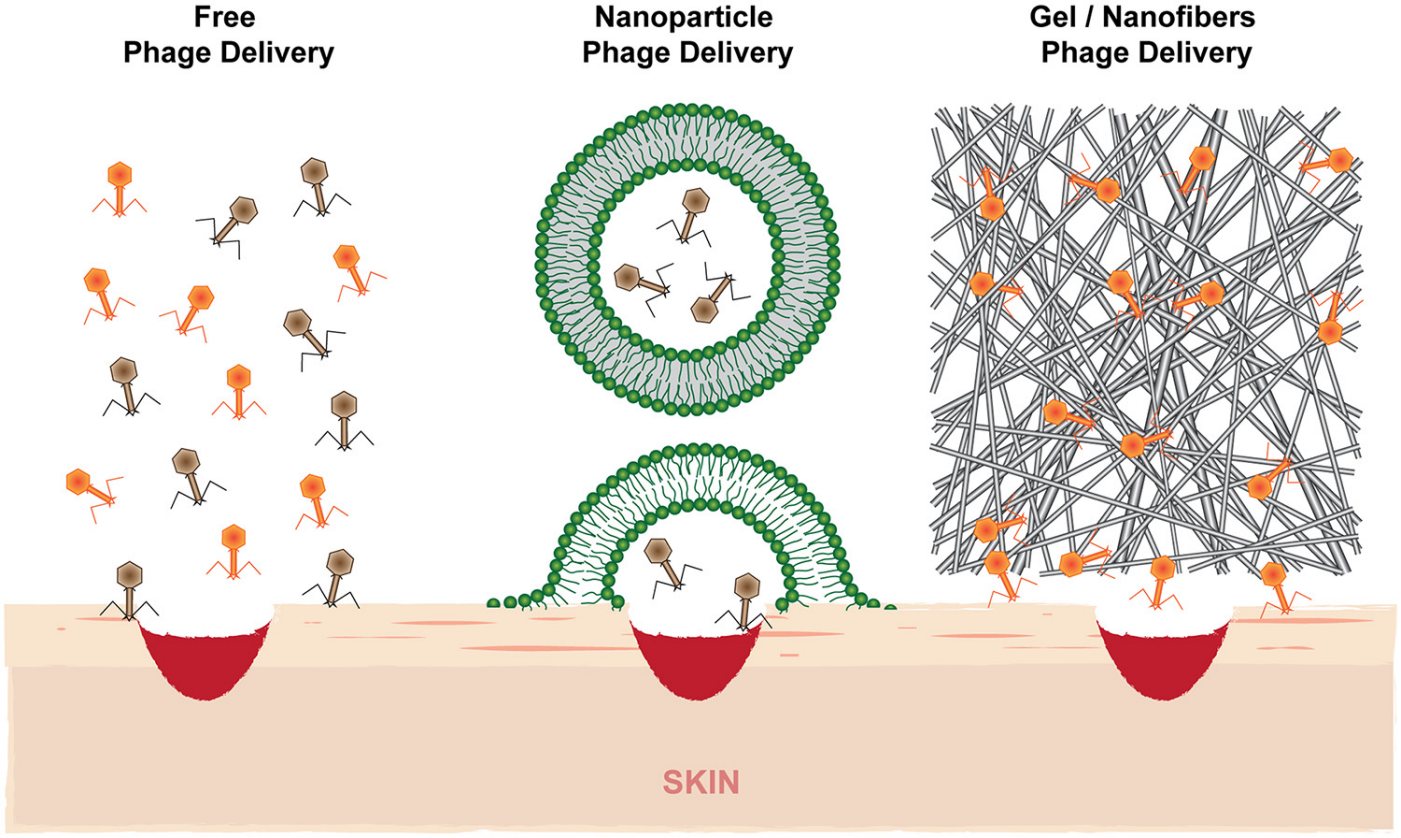
Topical delivery of phages via delivery systems such as liposomes (middle) compared to free-phage administration (left) allows deeper penetration of particles into the site of infection. Encapsulation of phages in hydrogels or fibers also allows long-term release of active phage particles as they are embedded in a protective matrix (right).

One of the most commonly used strategies is the encapsulation of phages or their immobilization. Encapsulated phages, e.g., inside liposomes, show a number of advantageous therapeutic properties over the administration of free phages (Fig. 1). The aim of any encapsulation process is to produce particles that monodisperse, i.e., similar in size and other physicochemical properties, and do not aggregate during production or application. Also, the number of phages per encapsulation particle (termed “loading” during the production) should not vary. If the two abovementioned criteria are not met, accurate dosing is not possible. As a general principle, phage preparations serve several purposes as follows.

(1) Protection. Encapsulation using, e.g., liposomes, protects the cargo from enzymatic attack, hydrolysis (low pH), and inactivation by components of the immune system.

(2) Stability. As biological entities, phages are deactivated when their proteins and/or nucleic acids degrade. This is particularly important for their storage.

(3) Active site delivery. The use of liposomes or detergent-lipid particles allows the penetration of the encapsulated cargo into the tissue, which often cannot be achieved when using free compounds.

(4) Availability. Fibers and hydrogels are a way of embedding phages in a three-dimensional network, hence allowing a constant release of phages to the site of action.

(5) Adhesion. In particular, positively charged materials, such as cationic hydrogels or liposomes, allow higher mucoadhesiveness, prolonging residence and release at the active site.

**(i) Liposomes.** Liposomes are spherical nanoparticles surrounded by a lipid bilayer that contain an aqueous solution, in which the therapeutic is contained; in the case of hydrophobic or amphiphilic molecules, the substance is found in the membrane or at the interface, respectively (49–51). Liposomes are highly biocompatible and are fairly easy to produce, e.g., by thin-film methods but also by gel-assisted rehydration, inverse emulsion, or microfluidics (52–54). Liposomes and related particles are highly versatile, as they can be prepared as multi- or unilamellar vesicles of various sizes, and their composition can be adjusted to allow modulation of surface charge and all other factors to influence delivery and pharmacokinetics (55, 56). Liposomes of a desired size can be produced by sonication, extrusion through membranes, or microfluidics (57, 58). Yet, they can adhere to each other and even undergo fusion under certain conditions and therefore not retain their size. The production of liposomes of precise dimensions, that do not aggregate or fuse, are important e.g., when used for intravenous administration.

Liposomes have been shown to penetrate bacterial biofilms to access the site of infection, which is often a problem for conventional antibiotics (59, 60). Aside from this, liposome encapsulation helps to retain phages at the infection site compared to nonencapsulated ones. In a murine burn model, longer retention times of five liposome-encapsulated *Klebsiella* phages were observed, which also showed higher efficacy compared to that of free phages (61). Longer phage retention times were also observed in a murine S. aureus diabetic wound model with the use of two myoviruses encapsulated in liposomes for which 33% shorter healing times were also reported (62). Aside from increasing the circulation of phages inside the patient (or model), liposomes also protect their cargo from enzymatic and chemical degradation, e.g., by low pH. Thus, liposome formulations are ideally suited for gastrointestinal infections via oral delivery. In the stomach, the acidic pH leads to phage protein denaturation, while enzymes in the gut degrade phage particles (63–65). In chickens, three *Salmonella* phages were observed to be more stable in gastric fluid (*in vitro*) and hence determined to have a longer duration of efficacy; one myxovirus and two *Podoviridae* were protected from degradation when encapsulated inside cationic lipid particles, which additionally extended residence time in the animals (57). The positive charge of the liposomes that were produced by thin-film hydration is believed to increase mucoadhesiveness. Additionally, the use of cationic lipids also increased the rate of encapsulation to around 50% and allowed a better dispersion in solution. When the formulation was freeze-dried, the particles stayed infectious longer than nonencapsulated phages.

Liposomes and other particles composed of amphiphilic molecules have the advantage that one can incorporate ligands that interact with target cells, which may increase directed delivery. This is not uncomplicated, as a ligand has to either show amphiphilic properties or needs to be conjugated with a molecule that anchors it to the nanoparticle, such as a lipid or detergent molecule. Homogeneous incorporation of this ligand molecule, potentially also directional (with all or most ligands facing the outside of the nanoparticle), is not easily accomplished. It would be easier to incorporate charged lipids that then allow an electrostatic interaction with mucosal tissues or dissolved biomolecules (50); this might, however, not be advantageous in all cases, as it might decrease circulation times or result in nonspecific interaction with the phage. Here, net-neutral lipids may be more suitable. The incorporation of passivating chemicals that prevent interactions between biomolecules and that are also not recognized by the immune system, such as polyethylene glycol (PEG), might further reduce nonspecific interaction and increase circulation time in the patient (66, 67). The retention time in the body positively correlates with smaller-sized liposomes, i.e., the smaller the liposomes (or related particles), the longer they circulate in the system. Additionally, smaller-sized particles increase the likelihood of cellular uptake via endocytotic mechanisms and/or membrane fusion. If particle uptake and delivery of active cargo into the host cytoplasm is successful, intracellular pathogens can be inactivated by phages, such as strains of enteroinvasive Escherichia coli, *Listeria*, or *Mycobacterium*. Liposome-based delivery strategies have been used, for example, with the mycobacterium phage TM4 (68). While a promising strategy, encapsulation yields of phages inside lipoparticles are low or liposome sizes are difficult to control in using thin-film hydration, gel-assisted rehydration, or inverse emulsion. Such disadvantages for these techniques create a bottleneck for the production of liposome-encapsulated phages, presenting a challenge for large-scale industrial production. Advancements in other fields, such as microfluidic mixing, have shown promise, increasing encapsulation rates while allowing control of size and composition of the particles (69). While this approach seems to work well with certain types of phages, including some *Myoviridae* and *Podoviridae* targeting P. aeruginosa (70), several issues have been identified with other phages, including their aggregation or the undesired attachment of phages to the surface of liposomes (69). In such cases, a technical solution, excluding microfluidic encapsulation, might be required, or the careful optimization of production processes, such as lipid composition or the osmolarity of the solution that they are dispersed in, might affect binding and/or insertion of proteins and proteinaceous structures (71, 72). More research is required to identify suitable protocols and strategies to allow high-yield encapsulation without aggregation of virus particles or their unwanted interaction with the nanoparticle material, phenomena that have not been considered much in the past. To date, the observed obstacles, such as low encapsulation efficiencies, difficulties in controlling liposome size, and the loss of active phage during preparation, demonstrate that liposomes are not the perfect delivery vehicle. Therefore, rigorous testing is required to establish the suitability of a delivery vehicle in general, i.e., if liposomes can be used and which type of lipids may be suitable.

In parallel, alternatives to liposomes have to be explored, such as the so-called transferosomes, which are detergent-containing liposomes. Transferosomes have been employed for the phage treatment of S. aureus skin and soft tissue infections in a mouse model. Transferosomes showed better skin penetration and a higher degree of protection in soft tissue than a free-phage cocktail (73). Niosomes, which are comprised of nonionic surfactants and other amphiphilic molecules together with cholesterol (74), however, face similar challenges as with all amphiphilic vesicle-like particles.

**(ii) Hydrogels.** Hydrogels are one of the most common materials extensively used in tissue engineering as polymer scaffolds, filling agents, or as delivery vehicles for biomolecules. Phage delivery via hydrogels can be achieved by encapsulating phages in a polymer or by immobilizing phages on solid supports. Phage hydrogel encapsulation offers several advantages and has been extensively studied. An example is *Staphylococcal* phage K, which showed high antibacterial activity in an alginate encapsulation and was effectively protected against the acidic stomach pH compared to free phage (75). A phage cocktail contained in alginate/CaCO_3_ microcapsules has also been produced for the treatment of broiler chickens infected with *Salmonella*. Similar to liposomes, a higher antibacterial activity of the encapsulated phages was observed when compared to that of the nonencapsulated phage cocktail (76). Chitosan-alginate bead encapsulation has prevented phage degradation during storage and allowed the phage titer of E. coli and Salmonella enterica phages to remain high in a gastrointestinal *in vitro* model, advocating for its use in the treatment or prophylaxis of intestinal pathogens of farm animals (77, 78).

Interestingly, immobilized phages do not activate the release of proinflammatory cytokines (such as interleukin-1α [IL-1α]) or stimulate antibody production, but they have been shown to be removed from systemic circulation into the liver and spleen of animal models where the phages remain active (79). This retention allows prolonged efficacy as blood circulation transports bacteria through the liver where the bacteriophages are trapped. Another fascinating use of such particles is for the uptake by immune cells, such as macrophages, which endocytosed 0.1-µm nylon nanoparticles coated with phages directed against intracellular S. enterica serovar Typhimurium strains, leading to efficient reduction or elimination of the pathogen (80).

**FIG 2:**
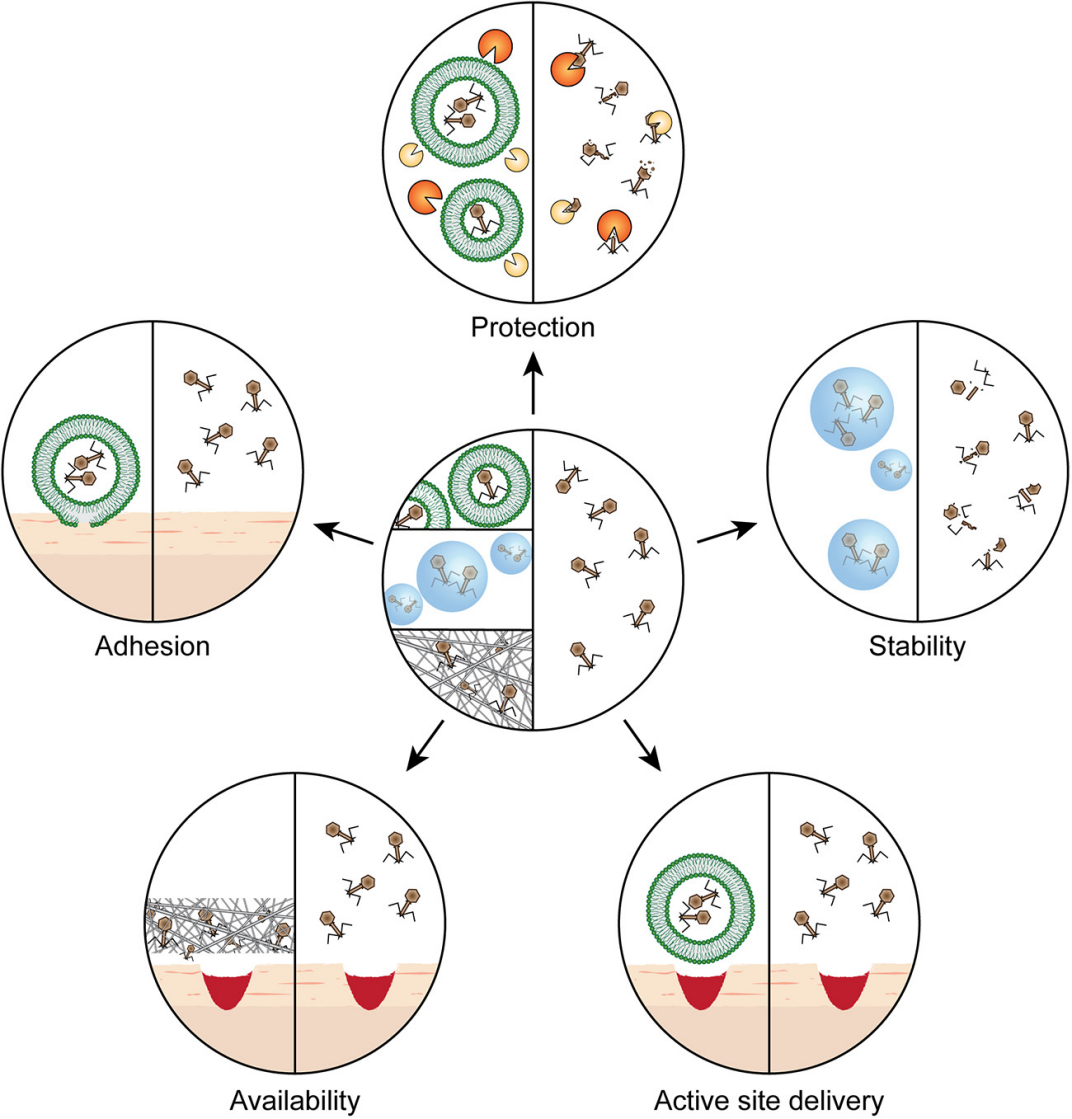
Properties of encapsulating phages for therapy versus the deployment of freely diffusing phages (center). (Clockwise from top) “Protection” from conditions that inactivate the phage, such as enzymes and acidic pH. The composition of the encapsulation material creates optimal conditions to secure “stability” during storage or administration of phages. “Active site delivery” is facilitated, e.g., by using liposome-encapsulated phages, which allow penetration into tissues. “Availability” is guaranteed when phages are embedded in a three-dimensional network, which retains the phage at the site of infection. “Adhesion” can be achieved by using suitable materials for encapsulation that allow interaction with the tissue.

In a recent study, polymerized fibrin glue was used as a *Pseudomonas* phage release carrier for local topical infections. This fibrin glue induced efficient bacterial lysis upon release of the phage particles from its matrix and is ideal for the prolonged topical delivery of phages (81). In a similar way, bacteriophages can be encapsulated in thin films, such as those generated from biocompatible material, such as whey protein isolate (WPI). As WPI-based films are very brittle compared to fibers, plasticizers like glycerol can be incorporated (82). This approach can be used to generate biocompatible coatings and has been demonstrated to allow prolonged storage of phages at ambient temperatures without significant loss of activity. When in contact with aqueous solutions, high concentrations of phage particles are released from the films, which then inactivate the target bacteria (83). Using a murine model, phages loaded onto polyvinyl alcohol-sodium alginate hybrid dressings were evaluated against S. aureus in burn wound infections and showed efficient antibacterial as well as wound-healing properties (84). In a recent study, an injectable bacteriophage-loaded hydrogel was shown to impede *in vitro* and *in vivo* P. aeruginosa colonization in treating local bone infections (85). Phages immobilized to hydrogel coating of silicone catheters have been shown to be efficient at preventing biofilm formation by E. coli, P. aeruginosa, Proteus mirabilis, K. pneumoniae, and Staphylococcus epidermidis in *in vitro* and *in vivo* models (86, 87). The efficacy of phage therapy can be maximized by employing suitable delivery methods (Fig. 2). Research in this field of study is still at its infancy, and novel delivery systems should be explored for the efficient delivery of phages to the site of infection.

**FIG 3:**
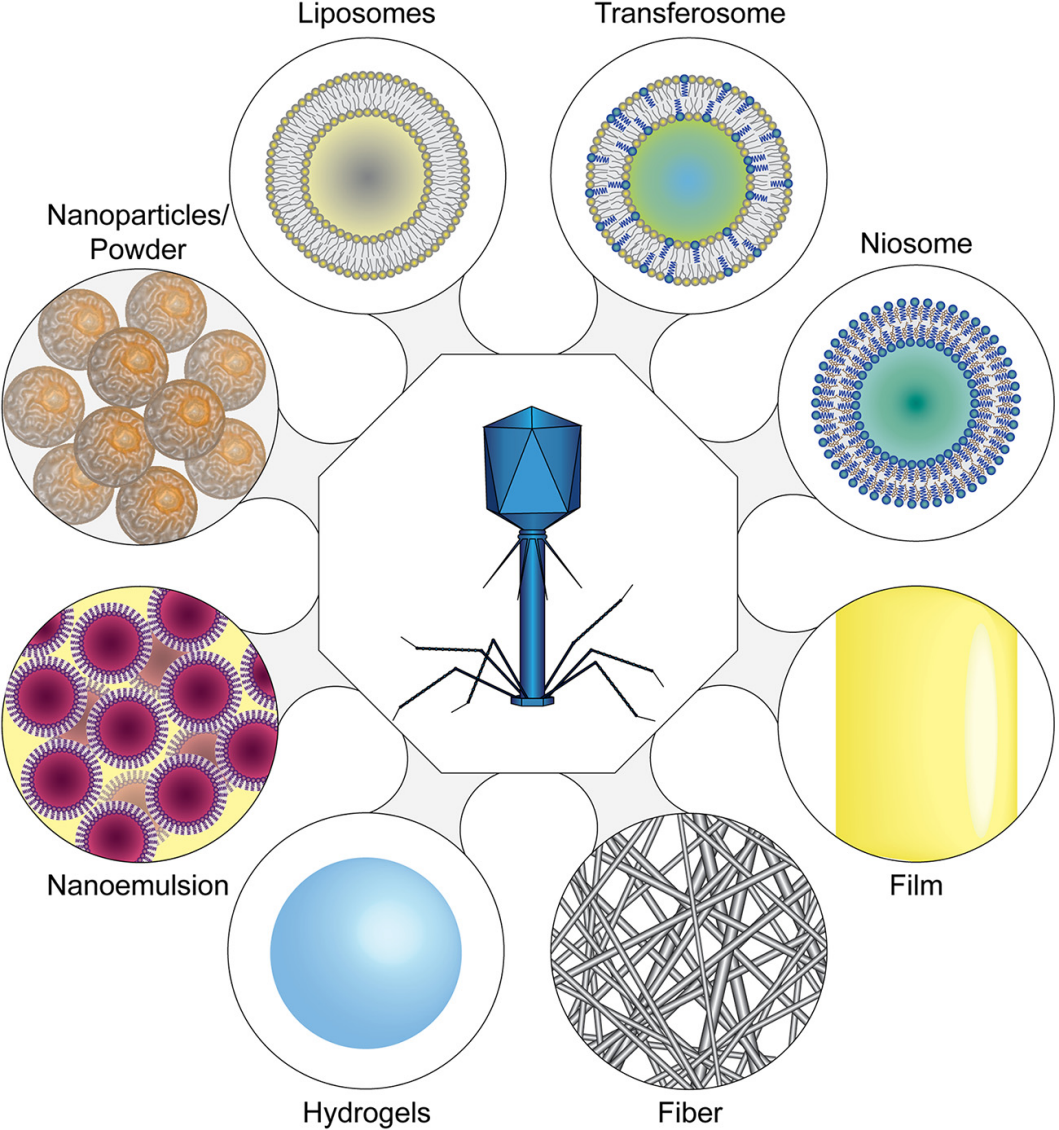
Phage encapsulation methods. (Clockwise from top) Liposomes, transferosomes, and niosomes represent small, aqueous “nanocontainers” that are separated from the outside by a barrier composed of amphiphilic molecules, which can be lipids (liposomes), lipid-detergent mixtures (transferosomes), or amphiphilic nonionic compounds together with cholesterol (niosomes). In contrast, films create a matrix in which bacteriophages are incorporated. Similarly, (nano-) fibers create a network of molecules that entrap the phages within yet still allow diffusion of the particles if fiber sizes permit. Hydrogels can create particles that allow the embedding of bacteriophages throughout the particle or larger objects such as films. Similar to films and fibers, phage particles are entrapped throughout the hydrogel network. Nanoemulsions are water droplets—which contain the phage—in an oil matrix with an emulsifying agent that prevents phase separation. Nanoparticles or larger powders may either contain phages within the compound that forms the particle matrix or present a surface to which the phages bind.

“Smart” systems or stimuli-responsive materials, i.e., systems that release embedded or immobilized bacteriophages upon a trigger, are particularly interesting. Such systems have been developed for long-term urinary catheters, where a pH-responsive surface coating allows the release of therapeutic bacteriophages when an infection occurs. Colonization by P. mirabilis can result in the formation of hard, crystalline biofilms blocking the catheter. The infection causes an increase in pH values of the urine; this triggers the release of phages from a pH-responsive surface hydrogel composed of the polymer poly(methyl methacrylate-co-methacrylic acid) (Eudragit S 100). In an *in vitro* bladder model system, the catheter blockage was delayed by a factor of 2 (65). Another class of “smart materials” is the thermo-responsive polymers, which undergo phase transition at distinct temperatures, allowing the release of therapeutic bacteriophages in infected wounds. Hathaway et al. developed nanospheres composed of poly-*N*-isopropyl-acrylamide copolymerized with allylamine, in which they incorporated the S. aureus phage K (88). The nanospheres were added to a nonwoven fabric, which can be used in adhesive bandages. At low temperatures, the phage particles remained embedded within the gel matrix. However, the nanospheres dissolved when temperatures elevated, which is generally observed at the site of bacterial skin infections, releasing active phage cargo and resulting in bacterial growth inhibition. A similar system that makes use of a double layer hydrogel has also been developed. Essentially, two layers of hydrogel were formed by coating an agarose gel containing the S. aureus phage K with hyaluronic acid (HA) methacrylate. During an infection, the HA outer layer is dissolved by enzymes produced by the pathogen and releases the phage in the vicinity of the infection (89). Figure 3 provides an overview of the various encapsulation methods developed thus far.

### Immobilization with fibers.

Apart from using “dry” particles (i.e., powders) and amphiphilic carriers (mainly, liposomes), phages can also be encased within or immobilized on surfaces. The generation of such “bioactive surfaces” not only benefits medicine and the food industry by targeting and inactivating bacterial pathogens, but also allows the detection, identification, and phage-mediated immobilization of target microbes. Surface immobilization of phages is an excellent strategy for the topical administration of phages in the form of wound dressings and bandages or as packaging material with antimicrobial properties in the food industry (90–92). Fibers, e.g., produced by electrospinning, have the advantages of being soft and flexible while at the same time being porous, thus exhibiting a large surface area. Phages, able to withstand an electric field as high as 40 kV/cm for 5 min, can be embedded into the fibers already during the electrospinning process, and a large variety of materials have been demonstrated to be suitable (cellulose diacetate [93], polyethylene oxide [93, 97], polyvinylpyrrolidone [94–96]). Compared to other materials, using fibers has the advantage of allowing tailored release of phage particles, which can be controlled by the choice of material. Different starting materials to create mixed-compound fibers or adjusting the molecular weight of the chemical building blocks allow researchers to tailor-make the kinetics of phage release (93, 97). Phage release is mediated by the swelling of fibers and/or disintegration of the material, either by so-called polymer erosion (by biological, chemical, or physical means) or simple dissolving of the polymers, if they are water-soluble.

Fiber production can expose phage particles to possible damage. As with the production of dry powders, rapid dehydration during the spinning procedure can inactivate phages and should, therefore, be avoided. Exemplary studies have been published with model phages, such as lambda, T4, and T7, where aqueous solutions of polyvinyl alcohol were used to prevent phages from dehydrating (98, 99). Additionally, the incorporation of sugars (e.g., trehalose) or the solvent composition can prevent phage inactivation by possibly stabilizing the phage and reducing the formation of salt crystals (95). While fibers can still be produced using pure distilled water, this composition is less than ideal for electrospinning, phage delivery, and long-term storage (95). Fibers comprised of a buffer solution instead have resulted in a morphology that has been shown to provide a thermodynamically favorable microenvironment for phages that will be encased within and hence retain phage infectivity over as many as 8 weeks (95).

Surface immobilization and the production of phage-embedded fibers that can be processed to fabrics or similar materials often face similar challenges during manufacture, as the starting point of both materials is, in many cases, a fiber-like structure. Rather than an encapsulation process occurring simultaneously with the production of the embedding matrix, another possibility is to immobilize phages on surfaces of finished materials. This postmanufacture embedding of phages onto fiber-based materials can be achieved by electrostatic means. Most tailed bacteriophages seem to exhibit a negative surface charge, allowing their interaction with positively charged materials such as alumina nanofibers (100), chemically modified silica (101), and polyvinyl-amine cellulose (90, 91). In addition to electrostatic binding, affinity-tag-mediated immobilization has also been used for the selective binding of phages, which display capsid protein-tag fusions. However, such an approach can negatively impact the biology of the modified phages (102).

In contrast to viruses that infect eukaryotic hosts, phages do not require cell uptake. Therefore, covalent binding strategies can also be employed using chemicals that allow cross-linking under mild conditions. Interestingly, bacteriophages that are covalently bound to a solid support are more heat stable than free phages, allowing sterilization by heat instead of radiation (79). Covalent binding has been explored for pathogen detection purposes, where phages were immobilized on chemically modified glass, gold, silica, carbon-nanotubes, and polymers of polyhydroxyalkanoate, polyethylene (PE), or cellulose (103–108). Other plastic polymers, such as polyethylene, polytetrafluoroethylene, and polycaprolactone (PCL), can be used for cross-linking phages to prevent the formation of bacterial biofilms in the clinic for catheters or implants (92, 109). Surgical threads that are composed of various polymers, including nylon, PE, and cellulose, have also been coated with phages (79, 80, 110, 111). A successful attempt to develop phage-based washable and nontoxic wound dressings made use of *Pseudomonas* bacteriophages covalently immobilized on the surface of polycaprolactone nanofibers and were shown to be effective even after 25 cycles of washing (92). In addition to phage-coated fiber-derived materials similar to electrospun materials, phages covalently bound to biodegradable polymers—poly(ester amide)s or polyester urea—can be prepared as wound dressings, with the possibility to embed additional substances, that are anti-inflammatory or pain-relieving, or chemical antibiotics (96, 112), while also containing enzymes that slowly degrade the material to allow the constant release of the substances into the wounds of patients (113–115).

## CONCLUSION

Globally, antibiotic-resistant bacterial infections are responsible for more than 750,000 deaths annually, and it has been estimated that mortality will reach approximately 10 million per year by 2050 (116). The future is looking bleak without other treatment options, as antibiotics are becoming increasingly ineffective; the study of the therapeutic potential of bacteriophages and the use of phage therapy as a standard clinical strategy to treat infections could be our way out of this crisis. Phages do have a promising potential to be used as therapeutic interventions in the treatment of antibiotic-resistant bacterial infections. However, there are still limitations that have to be addressed in order to allow phage therapy to become a standard strategy in clinical practice. One of them is the production of robust and reliable phage preparations, a critical issue. Pharmaceutical phage products need to fulfill many criteria, such as the issue of stability over long time spans and the suitability for delivery (i.e., nebulization) while also allowing targeted release, to only name a few. Due to their comparably unstable nature as biological entities, in particular, compared to small-molecule drugs, new pharmaceutical formulations might have to be developed for therapeutic phages. In recent years, advancements have been made in the field, and a plethora of options are readily available for the encapsulation and delivery of phages. While bacteriophages might not be able to replace chemical antibiotic compounds, the future will likely see a coexistence of both strategies, with phage therapy as an additional weapon against the bacterial world, possibly used in combination with antibiotics more often than on its own. To reach this status, however, robust preparation methods for the targeted delivery of therapeutic phages have to be established.
